# The Role of Immunotherapy in the Treatment of Malignant Pleural Mesothelioma

**DOI:** 10.3390/curroncol28060385

**Published:** 2021-11-08

**Authors:** Shantanu Banerji, Daniel E. Meyers, Craig Harlos, David E. Dawe

**Affiliations:** 1 CancerCare Manitoba Research Institute, Rady Faculty of Health Sciences, University of Manitoba, Winnipeg, MB R3E 0V9, Canada; ddawe@cancercare.mb.ca; 2 Department of Medicine, University of Calgary, Calgary, AB T2N 2T9, Canada; daniel.meyers@ucalgary.ca; 3 CancerCare Manitoba, Rady Faculty of Health Sciences, University of Manitoba, Winnipeg, MB R3E 0V9, Canada; charlos@cancercare.mb.ca

**Keywords:** immunotherapy, mesothelioma, nivolumab, ipilimumab, pembrolizumab, cell therapy

## Abstract

Malignant pleural mesothelioma is a rare and aggressive malignancy arising from mesothelial cells that line the serous membranes of the body. Cytotoxic chemotherapy has been a mainstay of therapy, resulting in a modest improvement in overall survival, but toxicity limits the eligible patient population. Few targeted agents beyond bevacizumab have demonstrated superior efficacy compared to placebos. With an improved understanding of the relationship between the immune system and cancer progression, immunotherapies are playing a greater role in the treatment of many cancers. Several early- and late-phase trials in malignant pleural mesothelioma, including assessments of the first-line efficacy of combination ipilimumab/nivolumab treatment, have now demonstrated promising results for both immune checkpoint inhibition and cell-based therapies. These immune therapies are likely to play a central role in the treatment of this disease going forward.

## 1. Introduction

Malignant pleural mesothelioma (MPM) is a rare but aggressive malignancy of which the incidence is highly correlated to the local importation and use of asbestos [1]. Incidence and mortality rates are increasing worldwide, with annual deaths estimated at 38,400 per year [2]. Optimal treatment can consider a multimodality approach, adding surgery and radiation to chemotherapy. However, a United States Surveillance, Epidemiology and End Results (SEER) database review of 14,228 cases diagnosed from 1973–2009 revealed that 59% of patients present with distant metastatic disease and only 23% of cases were treated with a cancer-directed surgery [3]. Median overall survival in this SEER population-based study was 7 months. Independent predictors of better survival include female sex, local disease stage at diagnosis, and younger age, whereas weight loss and sarcomatoid histologic subtype are associated with worse prognosis [4]. As in many other common cancers, cytotoxic chemotherapy is the traditional standard of treatment for patients with local or advanced MPM. However, with the loss of immune control now recognized as a “hallmark” of carcinogenesis [5] and the development of tolerable agents, immune-directed therapies are playing a greater role in the treatment of mesothelioma. The recently reported improved efficacy of a first-line combination of ipilimumab/nivolumab over standard platinum/pemetrexed treatment represents a breakthrough for immune therapies in the treatment of MPM.

## 2. Biology of Mesothelioma

MPM arises from the malignant transformation of normal mesothelial cells that line the serous membranes of the body, including the pleura, peritoneum, pericardium, and tunica vaginalis [6]. Although normal mesothelial cells are of a mesodermal origin, they express both mesothelial and epithelial markers [7]. Decades of chronic injury to the mesothelial lining ultimately leads to malignant transformation through a variety of postulated mechanisms, including the generation of toxic oxygen radicals in response to chronic inflammation, as reviewed in [6,8]. The ultimate diagnosis of MPM requires histologic confirmation either via percutaneous needle or surgical biopsy. Although patients often present with effusions at diagnosis, cytology alone is considered inadequate for initial diagnosis but can be used to assess disease recurrence or metastases [1]. The three most common histologic variants in MPM are epithelioid mesothelioma (~60% of new cases), sarcomatoid mesothelioma (~20%), and biphasic mesothelioma, which requires ≥10% of both epithelioid and sarcomatoid cell components to be present [1,6].

Genomic studies in MPM have revealed recurrent inactivating mutations, copy number loss, or gene fusions in several tumor suppressor genes, including ubiquitin carboxylterminal hydroxylase (BAP1), neurofibromin 2 (NF2), tumor protein 53 (TP53), large tumor suppressor kinase 2 (LATS2), SET domain-containing 2, histone lysine methyltransferase (SETD2), and cyclin-dependent kinase inhibitor 2A (CDKN2A) [9,10]. No recurrent activating mutations in oncogenes have been identified in MPM to date. However, downstream changes in key molecular pathways are being tested as rational strategies for novel targeted therapy in early-stage clinical trials, as reviewed previously [6]. In general, MPM has a lower protein-altering somatic mutation rate, especially compared to non-small-cell lung cancer (NSCLC) and small-cell lung cancer (SCLC). The mutation signatures most commonly seen in MPM indicate a base-agnostic mutagen (such as reactive oxygen species) and deamination of 5-methylcytosine to thymine in CpG islands [9]. Transversion mutations, commonly seen in association with exposure to cigarette smoke in lung cancer, are infrequent in mesothelioma.

MPM is associated with a diverse immune microenvironment consisting of tumor-associated macrophages (TAMS), cancer-associated fibroblasts, T-lymphocytes, and myeloid-derived suppressor cells, which contribute to MPM pathogenesis through complex autocrine and paracrine signaling, as reviewed in [8]. Despite the prominence of immune cells, many cells such as TAMS demonstrate an immunosuppressive phenotype, whereas cytotoxic T-lymphocytes often display positive immune checkpoint markers such as PD-1, TIM3, and LAG3, which are suggestive of functional exhaustion [8]. Cancer-associated fibroblasts contribute to both the disruption of immune cell dysfunction as well as the promotion of angiogenesis through the production of vascular endothelial growth factor (VEGF), among others. Transcriptomic analyses of MPM have revealed that the immune-checkpoint protein programmed cell death ligand 1 (PD-L1) is significantly overexpressed in the sarcomatoid subtype [9], whereas V-domain Ig suppressor of T cell activation (VISTA) is significantly overexpressed in epithelioid [10] mesothelioma. Cancer cells and other immune cells within the tumor microenvironment can express the B7 family protein PD-L1 or its corresponding receptor to trigger an adaptive immune response and avoid host immune-mediated destruction [11]. PD-L1 expression in MPM tumor cells is associated with worse overall survival but does not entirely predict the response to PD-1/PD-L1 inhibitors [8,12]. VISTA is expressed on antigen-presenting cells and impedes T cell responses by reducing proliferation and cytokine production [13].

## 3. Standard Systemic Therapy in Mesothelioma Prior to Immunotherapy

Historically, single cytotoxic drugs such as cisplatin, gemcitabine, or doxorubicin were considered the standard agents for the treatment of advanced MPM. In 2003, the multitargeted antifolate agent pemetrexed was studied in combination with cisplatin. At a dose of cisplatin at 75 mg/m^2^ and pemetrexed at 500 mg/m^2^ every 3 weeks, Vogelzang and colleagues demonstrated a statistically significant improvement in survival with first-line combination chemotherapy over single-agent cisplatin [14] (Table 1). Median overall survival (mOS) improved from 9.3 months to 12.1 months (hazard ratio (HR) 0.77, *p* = 0.02) with the combination over cisplatin alone. Patients received six cycles of therapy on average, with 5.3% of patients receiving eight or more cycles. An overall response rate (ORR) of 41.3% was observed on the combination arm, setting a new standard for systemic therapy in mesothelioma. Significant Grade 3/4 toxicities in the cisplatin/pemetrexed arm included leukopenia (40%), neutropenia (63%), nausea (33%) vomiting (30%), and fatigue (23%). The frequency of hematologic toxicity was reduced with the use of oral folic acid and intramuscular vitamin B12 supplementation. Similarly, the thymidylate synthesis inhibitor raltitrexed at 3 mg/m^2^ combined with cisplatin at 80 mg/m^2^ every 3 weeks improved mOS compared to cisplatin alone from 8.8 months to 11.4 months (HR 0.76, *p* = 0.048) [15]. With a median of five cycles, the ORR with combination therapy was 24% and Grade 3/4 toxicities were twice as common compared to monotherapy.

The outcomes for newly diagnosed advanced mesothelioma were further improved with the addition of the VEGF inhibitor bevacizumab to cisplatin/pemetrexed in the Phase III Mesothelioma Avastin Cisplatin Pemetrexed Study (MAPS). Bevacizumab at 15 mg/m^2^, when added to standard cisplatin/pemetrexed treatment, improved mOS from 16.1 months to 18.8 months (HR 0.77; *p* = 0.017) compared to placebo [16]. Seventy-five percent of patients in the experimental arm completed all six cycles of cisplatin/pemetrexed and a treatment benefit was observed regardless of age, sex, and histologic subtype. Although toxicity was reported to be manageable, the addition of bevacizumab led to an increase in the frequency of an any-grade creatinine concentration rise (10.6%), hemorrhage (33.8%), cardiovascular adverse events (59%), hypertension (55%), and arterial/venous thromboembolic events (5.9%) compared to placebo. Allowing for the limitations of a short-term follow-up, adding bevacizumab did not negatively impact patient quality of life. Although cisplatin/pemetrexed/bevacizumab promised to be a new standard of care in MPM, the combination has not been adopted universally across the globe [1]. With the success of the VEGF monoclonal antibody bevacizumab in combination therapy, the oral anti-angiogenic agent nintedanib was tested in combination with up to six cycles of cisplatin/pemetrexed in a Phase III trial. Nintedanib targets VEGF receptors 1–3, PDGF receptors alpha and beta, FGF receptors 1–3, and Src and Abl kinases. With a median duration of therapy of 5.3 months, nintedanib failed to meet its primary endpoint of improved median progression free survival (mPFS) compared to placebo (HR 1.01; *p* 0.91) [17].

The role of angiogenesis pathway inhibition in MPM remains unclear. Therefore, the standard of care for the first-line treatment of MPM has remained cisplatin/pemetrexed; however, bevacizumab can be considered in combination where accessible.

## 4. The Emerging Role of Immunotherapy in MPM

The last decade has presented a paradigm shift in the way we understand the relationship between the immune system, cancer development, and subsequent disease progression. Monoclonal antibodies directed against cytotoxic T lymphocyte antigen 4 (CTLA4) or programmed cell death 1 (PD-1) or its cognate ligand PD-L1 have received regulatory approval across the globe, alone or in combination with chemotherapy, for the treatment of a variety of malignancies, including other thoracic cancers such as NSCLC and SCLC [22,23,24,25].

### 4.1. Early-Phase Trials

The CTLA4 inhibitor tremelimumab was the first immune checkpoint inhibitor assessed in mesothelioma. Calabro and colleagues enrolled 29 patients with platinum-resistant disease on a Phase II trial of tremelimumab 15 mg/kg every 90 days until progressive disease or toxicity [26]. The median age was 64 with 86% of participants having epithelioid histology. Only two patients (6.9%) had an ORR and mOS was 10.7 months, with 36.7% surviving for 2 years. Grade 3/4 toxicity included colitis/diarrhea (13%), an increase in hepatic transaminase rise (6%), an increase in amylase (3%), and peripheral neuropathy (3%). A more intense schedule of intravenous tremelimumab (10 mg/kg every 4 weeks for seven doses, then every 12 weeks thereafter until treatment discontinuation) was compared to placebo in the randomized Phase IIb DETERMINE study [19] (Table 1). Patients with unresectable MPM who failed a platinum/pemetrexed regimen were randomized 2:1 to tremelimumab versus placebo. The median age was 66, 83% had epithelioid histology, and 69% had Stage IV disease. The median treatment duration with the CTLA4 inhibitor was 57 days and mOS was similar between groups at 7.7 and 7.3 months (HR 0.92, *p* 0.41) in the tremelimumab and placebo groups, respectively. An ORR was seen in only 4.5% of cases and the sarcomatoid subtype (accounting for 6% of cases) numerically seemed to benefit from the CTLA4 inhibitor more than the epithelioid subtype cases. No new adverse safety signal was observed.

The first study to assess the efficacy of the PD-1 inhibitor pembrolizumab was published in 2017 [27]. In the Phase 1b KEYNOTE-028 trial, Alley and colleagues enrolled patients with PD-L1 ≥ 1%-positive MPM—defined by membranous PD-L1 expression in 1% or more of the tumor and associated inflammatory cells, or positive staining in the stroma. Patients received pembrolizumab at 10 mg/kg intravenously every 2 weeks until disease progression, intolerable toxicity, or study withdrawal. Of 83 patients screened for enrollment via the testing of PD-L1 expression, 38 (46%) were positive and 25 were eligible for inclusion. Of these 25 patients, 92% were previously treated with cytotoxic chemotherapy and 72% had epithelioid histology. An ORR of 20% was observed (all partial responses) with a median duration of response of 12 months. Median overall survival was 18 months, with two patients completing the protocol-specified maximum 24 months of treatment. Treatment-related Grade 3 toxicity, observed in one patient each, included thrombocytopenia; dyspnea; iridocyclitis; alanine aminotransferase increase; and a combination of neutropenia, decreased appetite, and pyrexia (in the same patient). Multiple other Phase I/II non-randomized single agent PD-1/PD-L1 inhibitors studies including pembrolizumab, nivolumab, and avelumab have demonstrated an ORR of 19%–38% and an mOS of 7.2–17.3 months, as previously reviewed [28].

The modest but encouraging results with single-agent CTLA4 and PD-1/PD-L1 agents prompted combination trials to assess potential synergistic effects. The open-label Phase II NIBIT-MESO-1 study assessed treatment with tremelimumab at 1 mg/kg with durvalumab at 20 mg/kg every 4 weeks for four doses, followed by durvalumab alone for nine doses [29]. The tumor response was assessed using the modified Response Evaluation Criteria in Solid Tumors (mRECIST) for pleural mesothelioma, which measure tumor thickness perpendicular to the chest wall or mediastinum to determine the response. A total of 40 patients were assessed: with a median age of 64, 80% epithelioid histology, 73% Stage IV disease, and 30% as a first-line therapy. The ORR, as determined by mRECIST, was 25% and mOS was 16.6 months. Responses did not correlate with PD-L1 expression status and 18% of patients experienced Grade 3/4 immune-related toxicity, reversible with protocol guidelines. The French randomized open-label Phase II MAPS2 trial directly compared single-agent nivolumab at 3 mg/kg every 2 weeks to nivolumab plus ipilimumab at 3 mg/kg every 6 weeks until progression or toxicity in previously treated patients with advanced MPM [30]. A total of 125 patients were randomized to the two treatment arms: with a median age of 71, 84% epithelioid histology, and 69% in the second-line setting. The primary endpoint of disease control at 12 weeks was met in 44% of patients receiving nivolumab and 50% receiving the combination, which exceeded the prespecified target of 40%. Although an ORR was seen in 19% of nivolumab and 28% nivolumab/ipilimumab patients, disease hyper-progression (defined as >80% growth at 12 weeks) was seen in 10% and 4% of patients, respectively. mOS was 11.9 months with nivolumab and 15.9 months with the combination. Grade 3/4 adverse events were seen in 14% of patients on monotherapy compared to 28% in the combination arm. Studies of comprehensive immune cell profiling suggest that the PD-1/CTLA4 combination increases the activation and proliferation of effector memory T-cells compared to monotherapy [31].

### 4.2. Phase III Registration Trials of Immunotherapy in MPM

In the past year, three randomized Phase III studies have explored the role of PD-1 inhibitors alone or in combination with a CTLA4 inhibitor in advanced MPM [18,20,21]. The PROMISE-meso trial examined the role of pembrolizumab at a 200-mg fixed dose every 3 weeks compared to single-agent intravenous (IV) gemcitabine at 1000 mg/m^2^ (days 1 and 8) every 3 weeks or vinorelbine at 30 mg/m^2^ IV (days 1 and 8) or 60/80 mg/m^2^ oral (days 1 and 8) until progression. Cross-over to pembrolizumab was permitted in the chemotherapy arm [20]. A total of 144 patients who had progressed after previous platinum-based chemotherapy, stratified by histologic subtype, were randomized 1:1; 63% of patients randomized to single-agent chemotherapy subsequently crossed over to pembrolizumab. The median age was 70, with almost 90% having epithelioid histology. Although ORR with pembrolizumab was 22% compared to 6% with chemotherapy, this did not translate into any significant differences in mPFS or mOS even when correcting for cross-over or stratifying by PD-L1 expression status. Rates of treatment-related adverse events were similar between both arms. In early 2021, the results of the Phase III CONFIRM trial were presented at the 2020 World Conference on Lung Cancer [21]. After stratifying for epithelioid versus non-epithelioid histology, 332 MPM patients who progressed on one or more previous lines of chemotherapy were randomized 2:1 to nivolumab at a fixed dose of 240 mg IV every 14 days or placebo IV solution. Co-primary endpoints were overall survival and investigator-reported mPFS. The median age was 70, with 88% with epithelioid histology, and 57% treated in the 3rd line setting. Median OS was significantly improved at 9.2 months on nivolumab versus 6.6 months on placebo (HR 0.72, *p* 0.018) and was independent of PD-L1 expression status, but subgroup analysis suggested that patients with epithelioid histology derived a greater survival benefit. Rates of any-grade or ≥Grade 3 side effects were similar between nivolumab and placebo. Based on these results, single-agent nivolumab can be considered an option for MPM patients who have failed previous chemotherapy.

Finally, the CheckMate 743 randomized, open-label, Phase III study directly compared the global standard platinum/pemetrexed to nivolumab plus ipilimumab as a first-line therapy for advanced MPM [18]. The control arm received standard cisplatin/pemetrexed dosing for up to six cycles. Carboplatin (area under the curve: 5 mg/mL/min) could be substituted for cisplatin. The experimental arm received nivolumab plus ipilimumab at similar doses to the MAPS2 trial and treatment was permitted for up to 2 years. The primary endpoint was overall survival. A total of 605 patient were randomized 1:1, stratified by sex and histology to chemotherapy or immunotherapy, with a median age of 69, 75% epithelioid histology, 12% sarcomatoid histology, and ~50% Stage IV disease. PD-L1 status was quantifiable in 97% of patients and 77% of cases showed staining in ≥1% of tumor cells. Sixty-two percent of chemotherapy patients completed all six cycles of therapy and the median duration of nivolumab/ipilimumab therapy was 5.6 months. The study met its primary endpoint with a statistically significant improvement in mOS at 14.1 months in the control arm and 18.1 in the immunotherapy arm (HR 0.74, *p* = 0.002). Upon disease progression, 44% of patients in the immunotherapy arm received subsequent systemic therapy, 43% of whom received chemotherapy. In the chemotherapy arm 41% received subsequent therapy: 20% received immunotherapy and 31% received alternate chemotherapy. 

Although mOS with nivolumab/ipilimumab was similar in epithelioid and non-epithelioid patients, the latter group derived significantly less benefit with chemotherapy with mOS of 16.5 versus 8.8 months, respectively. Objective response rates with immunotherapy were 40%, similar to that of chemotherapy both in the MAPS2 trial and historical clinical trials of platinum/pemetrexed. PD-L1 positivity did not correlate with the degree of benefit. As with many studies in which immunotherapy is compared to chemotherapy, mPFS initially appears worse in part due to hyper-progression on nivolumab/ipilimumab, but numerically better at landmark time points (e.g., 12 months at 30% and 24%, respectively). Grade 3/4 adverse events were seen in 30% of the immunotherapy group and 32% receiving chemotherapy, although the rates of treatment-related Grade 3/4 serious events were greater in the nivolumab/ipilimumab arm (15% versus 6%). On the strength of these results, the United States Food and Drug Administration approved nivolumab in combination with ipilimumab for the first-line treatment of adults with unresectable malignant pleural mesothelioma in October 2020 and the European Medicines Agency adopted a favorable opinion in 2021 [32,33].

### 4.3. First-Line Immunotherapy in Combination with Chemotherapy

The results of treatment with immune checkpoint inhibitors are challenging existing chemotherapy standards of care and pose an efficacious first-line option, without an excess of adverse safety signals, especially for chemo-resistant sarcomatoid histologic subtypes. However, with response rates still around 40% overall, there is the possibility that immunotherapy in combination with chemotherapy may improve patient outcomes even further and avoid hyper-progression on immune checkpoint inhibitors, as seen in NSCLC and SCLC [23,24]. The PrE0505 Phase II single-arm study combined six cycles of platinum/pemetrexed with durvalumab followed by maintenance durvalumab for up to 1 year [34]. Median OS for the 55 treated patients was 20.4 months with an ORR of 56%. Grade 3 or higher adverse events occurred in 65% of patients with most related to chemotherapy. Durvalumab, pembrolizumab, or atezolizumab in combination with chemotherapy ± bevacizumab are currently being compared to standard-of-care chemotherapy in Phase III randomized control trials, with results expected as early as 2022 (Table 2).

### 4.4. Immunotherapy Strategies beyond Current Immune Checkpoint Inhibitors

Current therapeutic advancements with PD-1/PD-L1/CTLA4 inhibitors are encouraging but do not appear to be effective in all patients with MPM. A common theme in the existing immune checkpoint inhibitor approach is the requirement of an already primed immune microenvironment, specifically with the presentation of tumor antigens by antigen-presenting cells (APC) and activated T-cell mediated cytotoxicity [35]. The failure of adequate APC function or exhaustion of T-cell cytotoxic activity can therefore ultimately impact this response. Alternate immune checkpoints such as TIM-3 or LAG-3 are overexpressed in mesothelioma-associated T-lymphocytes and the combination blockade of these markers along with PD-L1 is showing promise in preclinical models [36]. Similarly, selectively targeting immunotoxins to mesothelin, a cell surface protein that is commonly expressed in mesothelioma, appears to enhance the effect of PD-1 inhibition [37].

Cellular therapy has been proposed as another novel approach to overcome the immunosuppressive microenvironment in MPM. Dendritic cell therapy (DCT) aims to expand the population of tumor-specific APC to generate a T-cell response. In brief, both a tumor cell lysate and peripheral blood mononuclear cells are obtained from a patient. The latter is enriched ex vivo to generate mature dendritic cells (DCs). Both autologous tumor lysate and DCs are then reinjected into the patient in order to trigger a tumor antigen-specific T-cell response [38]. A combined analysis of three studies with 29 MPM patients treated with DCT between 2006 and 2015 demonstrated an mOS of 27 months and a 5-year overall survival of 20.7% [39]. This approach is being tested against best supportive care in 230 participants through the Phase III DENdritic Cell Immunotherapy for Mesothelioma (DENIM) trial and the results are expected to be report in 2023 (NCT03610360).

Chimeric antigen receptor (CAR)-T cell therapy aims to address the issue of T-cell exhaustion. In brief, T-cells are extracted from patient peripheral blood and then genetically engineered to express a tumor-associated antigen-specific chimeric antigen receptor on their cell surface and expanded ex vivo. Engineered CAR-T cells undergo autologous re-injection into the patient and can identify specific tumor antigens without the requirement of an APC. Mesothelin-targeted CAR-T therapy in combination with pembrolizumab has demonstrated disease control [40]. Several early-stage trials are underway, as reviewed elsewhere [41], but likely require several more years of optimization before more widespread use.

Finally, oncolytic viral therapy can also be used to generate a disease-specific immune response when injected directly into the tumor, especially when modified to express immunogenic protein-like interferon-α or -β [42]. Early studies have demonstrated potential evidence of disease benefits and this strategy is currently being tested in the Phase III INFINITE clinical trial of recombinant adenoviral interferon combined with celecoxib and gemcitabine in MPM (NCT03710876).

## 5. Conclusions

Over the past 20 years, new agents have expanded the treatment compendium and expected survival for patients with advanced malignant pleural mesothelioma. Immune checkpoint inhibitors now pose a viable alternative to cytotoxic chemotherapy in many patients, either in treatment-naïve patients or as a subsequent line of therapy. Advances in cellular therapies also provide further opportunities to harness the immune system in the treatment of this disease. The optimal timing and combinations of these therapies are still being defined to maximize benefits but present an exciting future in the treatment of this challenging disease.

## Figures and Tables

**Table 1:** Key randomized trials in advanced malignant pleural mesothelioma.

Reference	Trial Phase	Line of Therapy	Histologic Breakdown	PDL1 ≥1%	Control and Experiment Arms	Sample Size	ORR, %	DCR, %	mPFS, Months	mOS, Months	Hazard Ratio
Non-Immunotherapy Trials											
Vogelzang, 2003 [14]	III	1st	68.3% E 25.9% NE	NR	Cisplatin Cisplatin/Pemetrexed	222 226	16.7 41.3	NR NR	3.9 5.7	9.3 12.1	0.77 *p* 0.02
van Meerbeeck, 2005 [15]	III	1st	67.6% E 24.4% NE	NR	Cisplatin Cisplatin/Raltitrexed	124 126	14 24	56.4 66.7	4.0 5.3	8.8 11.4	0.76 *p* 0.048
Zalcman, 2016 [16]	III	1st	81% E 19% NE	NR	Cisplatin/Pemetrexed Cisplatin/Pemetrexed/ Bevacizumab	225 223	NR NR	NR NR	7.3 9.2	16.1 18.8	0.77 *p* 0.017
Scagliotti, 2019 [17]	III	1st	96% E 4% NE	NR	Cisplatin/Pemetrexed Cisplatin/Pemetrexed/ Nintedanib	229 229	43 45	93 91	7.0 6.8	16.1 14.4	1.12 *p* 0.54
Immunotherapy Trials											
Baas, 2021 [18]	III	1st	75% E 25% NE	77%	Platinum/Pemetrexed Nivolumab/Ipilimumab	302 303	43 40	85 77	7.2 6.8	14.1 18.1	0.74 *p* 0.002
Maio, 2017 [19]	IIb	2nd (63%) 3rd (37%)	83% E 16% NE	NR	Placebo Tremelimumab	189 382	1.1 4.5	21.7 27.7	2.7 2.8	7.3 7.7	0.92 *p* 0.41
Popat, 2020 [20]	III	2nd	89% E 11% NE	46%	Gemcitabine or Vinorelbine Pembrolizumab	71 73	6 22	38 45.2	3.4 2.5	11.7 10.7	1.04 *p* 0.85
Fennell, 2021 [21]	III	2nd (30%) 3rd (57%)	88% E 12% NE	24%	Placebo Nivolumab	111 221	NR 10.4	NR NR	1.8 3.0	6.6 9.2	0.72 *p* 0.018

Abbreviations: PDL1, programmed death ligand 1; ORR, overall response rate; DCR, disease control rate; mPFS, median progression free survival; mOS, median overall survival; E, epithelioid; NE, non-epithelioid; NR, not reported; platinum, carboplatin, or cisplatin.

**Table 2:** Ongoing Phase III chemotherapy combined with immune checkpoint inhibitor trials in advanced malignant pleural mesothelioma.

ClinicalTrials.gov Identifier	Acronym	Trial Phase	Estimated Enrollment	Control Arm	Experimental Arm	Primary Endpoint	Estimated Primary Completion Date
NCT02784171	CCTG-IND227	III	520	Cisplatin + Pemetrexed	Cisplatin + Pemetrexed + Pembrolizumab	Overall survival	July 2022
NCT03762018	BEAT-meso	III	400	Carboplatin + Pemetrexed + Bevacizumab	Carboplatin + Pemetrexed + Bevacizumab + Atezolizumab	Overall survival	January 2024
NCT04334759	DREAM3R	III	480	Cisplatin/Carboplatin + Pemetrexed	Cisplatin/Carboplatin + Pemetrexed + Durvalumab	Overall survival	April 2025

Obtained from ClinicalTrials.gov, 15 September 2021.
